# The antibacterial activity and mechanism of ginkgolic acid C15:1

**DOI:** 10.1186/s12896-016-0324-3

**Published:** 2017-01-14

**Authors:** Zhebin Hua, Caie Wu, Gongjian Fan, Zhenxing Tang, Fuliang Cao

**Affiliations:** 1 Co-Innovation Centre for Sustainable Forestry in Southern China, Nanjing Forestry University, Nanjing, 210037 China; 2 College of Light Industry Science and Engineering, Nanjing Forestry University, Nanjing, 210037 China; 3 College of Forestry, Nanjing Forestry University, Nanjing, 210037 China

**Keywords:** GA, Green fluorescent protein, Antibacterial activity

## Abstract

**Background:**

The present study investigated the antibacterial activity and underlying mechanisms of ginkgolic acid (GA) C15:1 monomer using green fluorescent protein (GFP)-labeled bacteria strains.

**Results:**

GA presented significant antibacterial activity against Gram-positive bacteria but generally did not affect the growth of Gram-negative bacteria. The studies of the antibacterial mechanism indicated that large amounts of GA (C15:1) could penetrate GFP-labeled *Bacillus amyloliquefaciens* in a short period of time, and as a result, led to the quenching of GFP in bacteria. In vitro results demonstrated that GA (C15:1) could inhibit the activity of multiple proteins including DNA polymerase. In vivo results showed that GA (C15:1) could significantly inhibit the biosynthesis of DNA, RNA and *B. amyloliquefaciens* proteins.

**Conclusion:**

We speculated that GA (C15:1) achieved its antibacterial effect through inhibiting the protein activity of *B. amyloliquefaciens.* GA (C15:1) could not penetrate Gram-negative bacteria in large amounts, and the lipid soluble components in the bacterial cell wall could intercept GA (C15:1), which was one of the primary reasons that GA (C15:1) did not have a significant antibacterial effect on Gram-negative bacteria.

## Background

Plants can synthesize over 200,000 compounds through various metabolic pathways [1]. Secondary metabolites in plants are derived from primary metabolites, and their categories and chemical structures are complex and diverse, including nitrogen-containing organic compounds, terpenoids, phenols and polyacetylenes, of which alkaloids, terpenoids and phenols are the most common. Secondary metabolites are widely involved in plant growth, development and defense as well as other physiological and biological processes [2]. Plant secondary metabolites provide many useful natural organic compounds for human use. Because traditional chemical pesticides contaminate soil and water, the development of environmentally friendly bio-pesticides has become a popular research focus. However, the development of synthetic pesticides has many problems, such as a low successful rate, long cycle and huge cost etc. Therefore, discovering lead compounds (plant-derived antibacterial reagents) from natural plant products with improved biological activity has become an effective method to develop new biological pesticides. Self-defense mechanisms have been evolved in plants, and many secondary plant metabolites are natural antibacterial agents [3–5]. Wilkins et al. [6] reported that 1389 plants could be used as sources of plant antibacterial agents including ingredients that could kill or inhibit bacteria, such as antibiotics, flavonoids, organic acids, polyphenols and specific proteins. Wilson et al. [7] studied the inhibition of *Botrytis cinerea* by 345 crude plant extracts and 49 essential oils, found that 13 crude extracts and 4 essential oils provided antibacterial activities.

Resorcinolic lipids are widely distributed plant secondary metabolites produced in large numbers. Recent studies have shown that they have extraordinarily high antibacterial activity. Resorcinolic lipids produced by *Pseudomonas carboxydoflava* can inhibit the growth of many bacteria species, such as *Micrococcus lysodeictius* and *Bacillus subtilis* [8, 9]. Resorcinolic lipids isolated from cashew apple have strong antibacterial effects on Gram-positive bacteria, including methicillin-resistant *S. aureus* strains [10, 11]. Sixteen phenolic compounds have been isolated from the cashew *Anacardium occidentale* (Anacardiaceae) nut shell oil, including various C15 phenolic compounds. Their antimicrobial activity has been tested against four typical microorganisms, *Bacillus subtilis*, a Gram-positive bacterium; *Escherichia coli*, a Gram-negative bacterium; *Saccharomyces cereuisiae*, a yeast; and *Penicillium chrysogenum*, a mold. Most of them exhibited potent antibacterial activity against only Gram-positive bacteria [12].

Ginkgo is a Chinese-specific rare relict species that is well known as a “living fossil of gymnosperms” [13]. The fruit and leaves of ginkgo have relatively high economic and medicinal values. However, its sarcotestas is usually discarded, causing secondary pollution of the environment [14]. GA, which is in high level in sarcotestas, is a natural plant-derived active substance contained in ginkgo, and it belongs to long-chain phenolic compounds that are derivatives of sumac acid [15]. Current studies have shown that the biological activities of GA include anti-tumor activity, neuroprotective activity, anxiolytic and antibacterial activity [16–20]. These biological activities may make a possibility that increases the utilization of ginkgo sarcotestas and reduces environmental pollution. The potential uses of Ginkgo have been attracted many concern. Studies of GA antibacterial activity have found that although GA could inhibit the activity of bacteria and plant pathogens, it just showed selective antibacterial activity, with strong inhibition towards to Gram-positive bacteria and almost no inhibition to Gram-negative bacteria [21–24].

The present study employed GFP-labeled strains and analyzed the antibacterial activity and mechanisms of GA C15:1 monomer, high amounts of which was in ginkgo sarcotestas and had relatively high antibacterial activity. Investigations of the selective antibacterial activity of GA could provide a scientific and theoretical basis for the development of new plant-derived pesticides using ginkgo sarcotestas as the raw material.

## Results

### Antibacterial activity of GA (C15: 1)

The antibacterial activity of GA (C15:1) is shown in Table 1. GA (C15:1) had strong antibacterial activity against Gram-positive bacteria, the MIC values of all of the tested Gram-positive bacteria were not greater than 10 μg mL^−1^..In this study, all of the tested Gram-negative bacteria could grow well after the addition of large doses of GA (C15:1) (final concentration 500 μg mL^−1^), and no differences were observed compared with the controls supplemented with salicylic acid, indicating that GA (C15:1) did not have significant antibacterial action against Gram-negative bacteria.

**Table 1:** Antibacterial activities of GA (C15:1) and salicylic acid

Strains		Control		GA (C15:1)		Salicylic acid	
		MIC (μg mL^−1^)	MBC (μg mL^−1^)	MBC (μg mL^−1^)	MBC (μg mL^−1^)	MIC (μg mL^−1^)	MBC (μg mL^−1^)
G^−^	*E. coli* DH5α	>500	-	>500	-	>500	-
	*E. coli* O157:H7	>500	-	>500	-	>500	-
	*P. putida* KT2440	>500	-	>500	-	>500	-
	*P. aeruginosa* PAO1	>500	-	>500	-	>500	-
	*R. solanacearum*	>500	-	>500	-	>500	-
G^+^	*B. amyloliquefaciens* SQR9	>500	-	5	60	500	>500
	*R. jostii* RHA1	>500	-	10	20	500	>500
	*S. thermophilus* ND03	>500	-	10	20	500	>500
	*S. aureus*	>500	-	10	20	500	>500

*-* Not measured, *GA* ginkgolic acid, *MIC* the minimum inhibitory concentration, *MBC* the minimum bactericidal concentration

### The effect of GA (C15:1) on GFP in bacteria

**Fig. 1:**
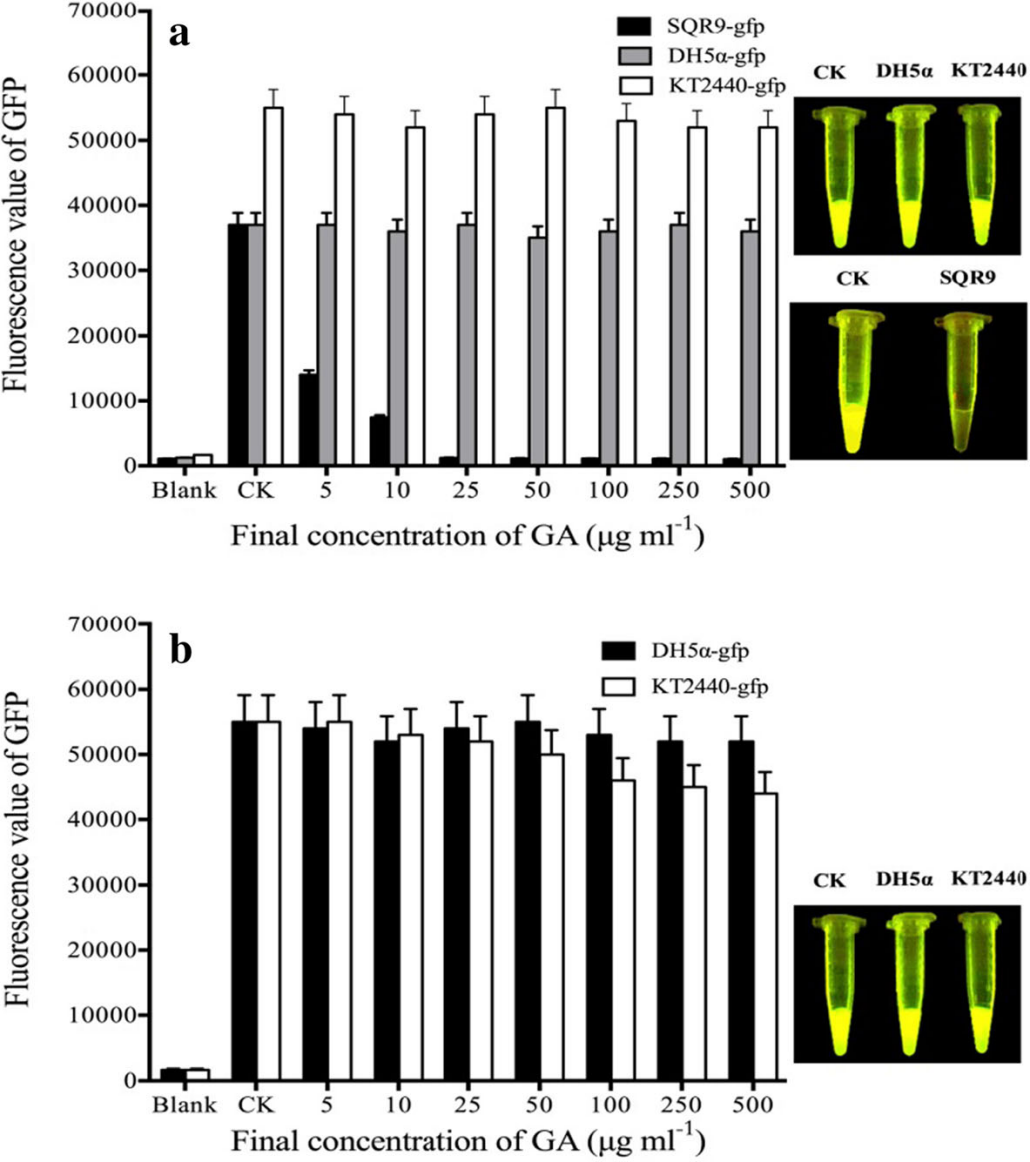
Effect of GA (C15:1) on GFP fluorescence in bacteria. Three independent experiments were conducted (*n* = 3); the error bars indicate one standard error. Three individual tubes were collected from LB plates, and each tube was performed in triplicate. Each bar in the gram represents means of three individual tubes (mean + − non-log transfoemed SE.). **a**: Bacteria were incubated at 30 °C for 1 min before GFP fluorescence was measured.10 μL of DMSO without the drug was used as a control. The blank was the *E. coli* bacteria solution without GFP. One-way ANOVA was used for analyzing the data (F_7,16_ = 656.9 *P* < 0.001(SQR9-gfp); F_7,16_ = 0.208 *P* > 0.05(DH5α-gfp); F_7,16_ = 0.357 *P* > 0.05 (KT2440-gfp)); (**b**) Bacteria were incubated at 30 °C for 4 h before GFP fluorescence was measured. 10 μL of DMSO without the drug was used as a control. The blank was the *E. coli* bacteria solution without GFP. One-way ANOVA was used for analyzing the data (F_7,16_ = 0.178 *P* > 0.05(DH5α-gfp); F_7,16_ = 1.412 *P* > 0.05 (KT2440-gfp))

Using a GFP-labeled strain as the target, we studied effect of GA (C15:1) on GFP fluorescence in bacteria, and the results are show in Fig. 1-a). GA (C15:1) could significantly affect GFP fluorescence in the Gram-positive bacteria *B. amyloliquefaciens* SQR9-gfp within 1 min. Compared with the results for the CK (bacteria only containing DMSO), GA (C15:1) at the concentration of 5 μg mL^−1^ could reduce GFP fluorescence intensity in SQR9 bacteria by more than 50% within 1 min, and GA (C15:1) at higher concentrations could almost completely quench GFP fluorescence in SQR9 bacteria within 1 min.

Although GA (C15:1) could significantly affect GFP fluorescence in *B. amyloliquefaciens* SQR9-gfp bacteria within 1 min, it did not have a significant effect on GFP fluorescence in Gram-negative bacteria *E. coli* DH5α-gfp and *P. putida* KT2440-gfp. Within 1 min, a significant decrease of fluorescence intensity was not detected in the studied Gram-negative bacteria, and fluorescence intensity values in the CK were close to the fluorescence intensity value in bacteria supplemented with GA.

We extended the contact time of Gram-negative bacteria *E. coli* DH5α-gfp and *P. putida* KT2440-gfp with GA (C15:1) to 4 h. The results (Fig. 1-b) showed that even with longer incubation times, GA could only reduce GFP fluorescence in Gram-negative bacteria by a small amount. GA (C15:1) at the concentration of 500 μg mL^−1^ had the most significant effect on fluorescence in *E. coli* DH5α-gfp, causing approximately 30% fluorescence reduction. The fluorescence reduction values at other concentrations were all less than 25%.

**Fig. 2:**
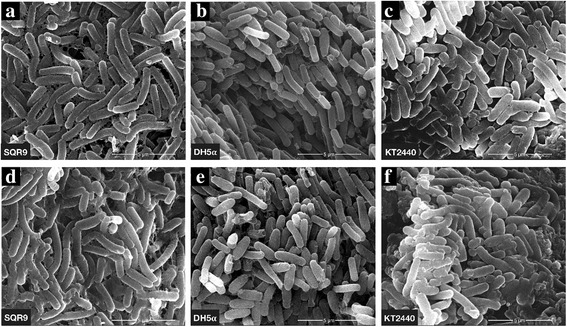
SEM observation of bacteria cell morphology. respectively, (**a**, **b**, **c**) represent Bacteria cell morphology before the addition of GA; (**d**, **e**, **f**) represent Bacteria cell morphology after the addition of GA at a final concentration of 100 μg mL^−1^ for 1 min

The scanning electron microscopy examination showed that after the addition of GA (C15:1), the cells of the three bacteria still remained intact without apparent cell lysis (Fig. 2). Because GFP protein was only present in the bacteria, we speculated that GFP fluorescence decay in Gram-positive bacteria *B. amyloliquefaciens* SQR9-gfp was caused by a large amount of GA that entered the bacteria within a short time, whereas the reason that GFP fluorescence in both Gram-negative bacteria did not show decay was that GA (C15:1) did not enter these bacteria in a large amount. The lack of a significant reduction in GFP fluorescence in the two Gram-negative bacteria was caused by a limited amount of GA entering the cells.

### Effect of GA (C15:1) on GFP in bacteria crude extracts

**Fig. 3:**
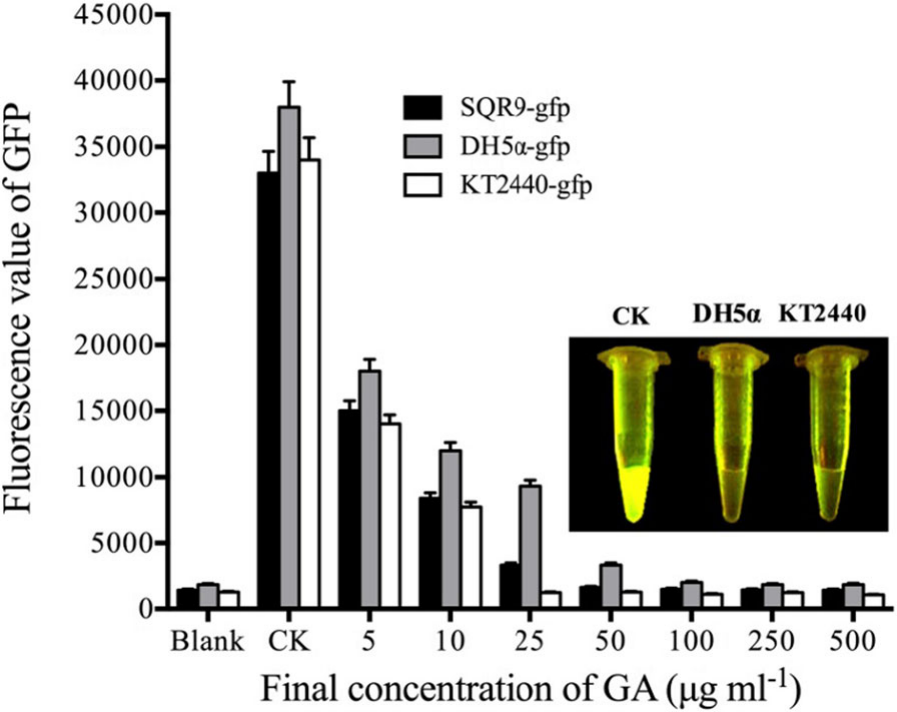
Effect of GA (C15:1) on GFP fluorescence in bacteria crude lysates (1 min). Three independent experiments were conducted (*n* = 3). The error bars indicate one standard error. All of the tests were incubated at 30 °C for 1 min before GFP fluorescence was measured. 10 μL of DMSO without the drug was used as a control. The blank was the *E. coli* bacteria solution without GFP. One-way ANOVA was used for analyzing the data (F_7,16_ = 281.4 *P* < 0.001 (SQR9-gfp); F_7,16_ = 246.3 *P* < 0.001 (DH5α-gfp); F_7,16_ = 304.0 *P* < 0.001 (KT2440-gfp)). Three individual tubes were collected from LB plates, and each tube was performed in triplicate. Each bar in the gram represents means of three individual tubes (mean + − non-log transfoemed SE.)

To verify the hypothesis that “GFP fluorescence decay was related to GA entering the bacteria cells”, the bacteria cells of Gram-negative bacteria *E. coli* DH5α-gfp and *P. putida* KT2440-gfp were lysed and centrifuged, and the crude lysate supernatants which contained GFP, were collected. GA (C15:1) was directly added to the supernatant, and the GFP fluorescence intensity was examined. The same procedure was performed on Gram-positive bacteria *B. amyloliquefaciens* SQR9-gfp. The results showed that GA (C15:1) could significantly affect GFP fluorescence in the crude lysates of *E. coli* DH5α-gfp and *P. putida* KT2440-gfp within 1 min (Fig. 3). Compared with the results for the CK, GA (C15:1) at a final concentration > 25 μg mL^−1^ could completely quench GFP fluorescence in the crude lysates of *E. coli* DH5α-gfp and *P. putida* KT2440-gfp within 1 min.

Similar fluorescence decay results were also showed in the crude cell lysates of Gram-positive bacteria *B. amyloliquefaciens* SQR9-gfp (Fig. 3). When the concentration of GA (C15:1) was > > 10 μg mL^−1^, the fluorescence in the crude lysates of *B. amyloliquefaciens* SQR9-gfp was completely quenched within 1 min. This results were consistent with the results of the GFP fluorescence decay experiment in *B. amyloliquefaciens* SQR9-gfp cells, and suggested that GA could enter the cells of Gram-positive bacteria *B. amyloliquefaciens* SQR9-gfp GFP within a short period of time.

### Effect of GA on the activity of a variety of proteins

**Fig. 4:**
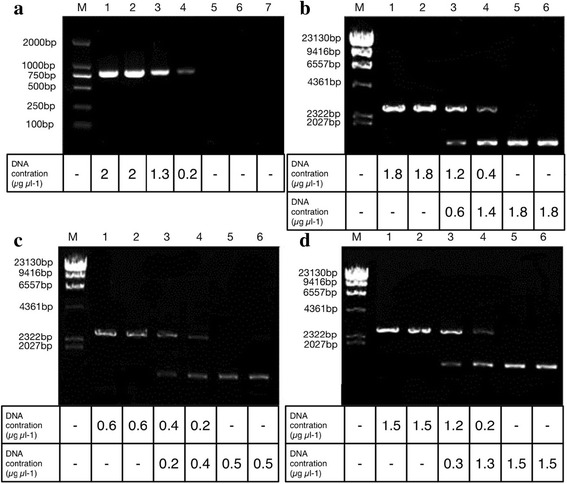
Effect of GA (C15:1) on a variety of proteins. respectively :(**a**) represents the effect of GA (C15:1) on the activity of *Taq* DNA Polymerase. The DNA contration at the bottom of fig represents the PCR reaction was inhibited by GA (C15:1). **b** represents the effect of GA (C15:1) on the activity of *Kpn* I. The DNA contration at the bottom of fig represents the enzymatic activity *Kpn* I was inhibited by GA (C15:1) (**c**) represents the effect of GA (C15:1) on the activity of *Hin*d III. The DNA contration at the bottom of fig represents the enzymatic activity *Hin*d III was inhibited by GA (C15:1) (**d**) represents the effect of GA (C15:1) on the activity of *Eco*R I. The DNA contration at the bottom of fig represents the enzymatic activity *Eco*R I was inhibited by GA (C15:1) lane 1, CK; lane 2, addition of DMSO into PCR or digestion system; lane 3, addition of 1 μg mL^−1^ GA (C15:1) into PCR or digestion system; lane 4, addition of 5 μg mL^−1^ GA (C15:1) into PCR or digestion system; lane 5, addition of 10 μg mL^−1^ GA (C15:1) into PCR or digestion system; lane 6, addition of 25 μg mL^−1^ GA (C15:1) into PCR or digestion system; lane 7, addition of 50 μg mL^−1^ GA (C15:1) into PCR or digestion system

According to the PCR results (Fig. 4-a), GA (C15:1) significantly inhibited the PCR reaction. The addition of 1 μg mL^−1^ and 5 μg mL^−1^ GA (C15:1) could interfere with PCR reactions, resulting in decreased specific bands, although these concentrations would not completely inhibit PCR reactions. However, when the concentration of GA was ≥ 10 μg mL^−1^, the PCR reaction was completely inhibited, and electrophoresis could not detect specific target bands. This result suggested that lower concentration of GA (C15:1) could inhibit Taq DNA polymerase activity and interfere with DNA replication.

Restriction digestion electrophoresis results showed that GA (C15:1) could significantly inhibit the enzymatic activity of Kpn I, Hind III and EcoR I (Fig. 4-b, c, d). GA (C15:1) at a final concentration of 1 μg mL^−1^ could partially inhibit the enzymatic activity of the three restriction enzymes. When the concentration of GA (C15:1) was > 5 μg mL^−1^, the enzymatic activity of the three restriction enzymes were inhibited almost completely, and the super coiled pUC19 plasmid was barely digested.

**Fig. 5:**
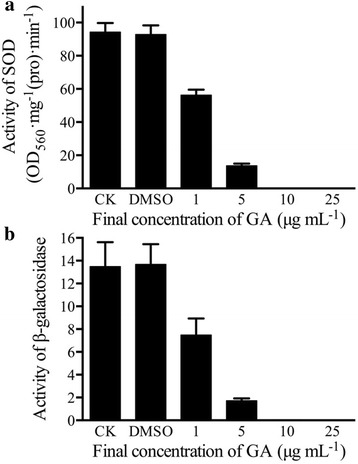
Effect of GA (C15:1) on the activity of SOD and β-galactosidase. The error bars indicate one standard error. **a**: Effect of GA (C15:1) on the activity of SOD.The optical density (OD)_560_ values were measured. 5 μL of DMSO (without the drug) was used as a control. One-way ANOVA was used for analyzing the data (F_5,12_ = 161.6 *P* < 0.001);(**b**) : Effect of GA (C15:1) on the activity of β-galactosidase. The OD value at 420 nm was read. 5 μL of DMSO (without the drug) was used as a control. One-way ANOVA was used for analyzing the data (F_5,12_ = 25.15 *P* < 0.001). Three individual tubes were collected from LB plates, and each tube was performed in triplicate. Each *bar* in the gram represents means of three individual tubes (mean + − non-log transfoemed SE.)

GA (C15:1) could significantly inhibit SOD enzyme activity and β-galactosidase activity (Fig. 5-a, b). When the final concentration of GA (C15:1) was 1 μg mL^−1^, both SOD enzyme activity and β-galactosidase activity were decreased by 50% compared with that of the control. When the final concentration of GA was > 5 μg mL^−1^, SOD enzyme activity and β-galactosidase enzyme activity were almost undetectable.

These proteins had different sources and were selected randomly. Thus, the results of this study suggested that the inhibition of GA (C15:1) on protein activities was non-selective.

### Inhibition of isotope incorporation experiments

**Fig. 6:**
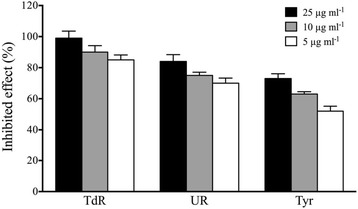
Effect of GA (C15:1) on the incorporation of precursors for the synthesis of macromolecules in *B. amyloliquefaciens* SQR9. The error bars indicate one standard error. The final concentrations of GA in the reaction system were 25 μg mL^−1^, 10 μg mL^−1^ and 5 μg mL^−1^, respectively. 5 μL of DMSO (without the drug) was used as a control. All of the treatments were conducted at 37 °C in a shaker. One-way ANOVA was used for analyzing the data (F_2,6_ = 8.72 *P* = 0.017 (TdR); F_2,6_ = 19.54 *P* = 0.002 (UR); F_2,6_ = 28.59 *P* < 0.001 (Tyr)). Three individual tubes were collected from LB plates, and each tube was performed in triplicate. Each bar in the gram represents means of three individual tubes (mean + − non-log transfoemed SE.)

The aforementioned experiments showed that GA could inhibit DNA polymerase function in vitro. However, it was unclear whether GA had a similar function in bacteria, including whether GA (C15:1) could inhibit DNA polymerase activity in vivo, which would inhibit DNA replication. In addition, it is unclear whether GA could inhibit RNA polymerase and ribosome activities, which would inhibit transcription and translation. To further clarify the mechanism of GA (C15:1), we used the method of the inhibition of isotope incorporation to verify the effects of GA (C15:1) in vivo. (Methy-^3^H) thymine ([^3^H] TdR), ^3^H-uridine ([^3^H] UR) and ^3^H-tyrosine ([^3^H] Tyr) were used as precursors to determine effect of GA (C15:1) on the biosynthesis of DNA, RNA and *B. amyloliquefaciens* SQR9 proteins. The results are showed in Fig. 6. Compared with that of the control, GA (C15:1) could inhibit DNA replication, RNA synthesis and protein synthesis to different extents under all three concentrations (25 μg mL^−1^, 10 μg mL^−1^ and 5 μg mL^−1^). When the concentration of GA (C15:1) reached 25 μg mL^−1^, the inhibition of [^3^H] TdR incorporation was approximately 99%, of [^3^H] UR incorporation was approximately 90%, and of protein precursor [^3^H] tyrosine was approximately 85%. These data indicated that GA (C15:1) could inhibit DNA replication in vivo as well as RNA transcription and protein synthesis.

### The interception of GA by Gram-negative bacteria cell walls

**Fig. 7:**
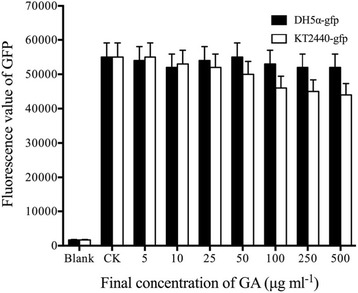
Effect of GA (C15: 1) on GFP fluorescence in the protoplasts of *E. coli* DH5α-gfp (1 min). Three independent experiments were conducted (*n* = 3); The error bars indicate one standard error. 5 μL of DMSO (without the drug) was used as a control. The blank was *E. coli* protoplast solution without GFP. All the tests were incubated at 30 °C for 1 min. One-way ANOVA was used for analyzing the data (F_7,16_ = 0.106 *P* > 0.05 (DH5α-gfp); F_7,16_ = 1.412 *P* > 0.05 (KT2440-gfp)). Three individual tubes were collected from LB plates, and each tube was performed in triplicate. Each bar in the gram represents means of three individual tubes (mean + − non-log transfoemed SE.)

Using *E. coli* as the target, lysozyme was used to destroy the peptidoglycan structure in the cell wall and thus *E. coli* DH5α-gfp protoplasts were obtained. Effect of GA (C15:1) on GFP fluorescence in the protoplast within 1 minute was measured. The results (Fig. 7) showed that when the final concentration of C15:1 was lower than 25 μg mL^−1^, it did not have significant effect on GFP fluorescence in protoplasts. When the final concentration of GA was ≥ 25 μg mL^−1^, the decreased GFP fluorescence intensity in the protoplasts was found. It was positively correlated with increasing concentration of GA. When the final concentration of GA reached 500 μg mL^−1^, the GFP fluorescence intensity in the protoplasts decreased to approximately 80% of the control. This result suggested that after the peptidoglycan structure in the Gram-negative bacteria cell wall was destroyed, a small amount of high concentration GA could enter the Gram-negative bacteria cell and produce a low level of GFP fluorescence decay.

**Fig. 8:**
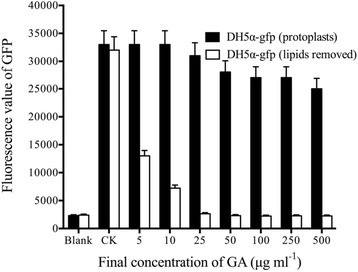
Effect of GA (C15: 1) on GFP fluorescence in *E. coli* DH5α-gfp (1 min), in which the lipids have been removed from the strains’ cell wall. Three independent experiments were conducted (*n* = 3). The error bars indicate one standard error. 5 μL of DMSO (without the drug) was used as a control. The blank was E. coli bacteria solution without GFP (after ethanol solubilization). All the tests were incubated at 30 °C for 1 min. One-way ANOVA was used for analyzing the data (F_7,16_ = 2.116 *P* > 0.05 (*Black*); F_7,16_ = 121.6 *P* < 0.001 (*White*)). Three individual tubes were collected from LB plates, and each tube was performed in triplicate. Each bar in the gram represents means of three individual tubes (mean + − non-log transfoemed SE.)

By soaking *E. coli* cells in ethanol solution for a short period of time, the lipid-soluble components (mainly included lipopolysaccharide and phospholipids) in the cell wall were removed/partially removed. Effect of GA (C15:1) on GFP fluorescence in the *E. coli* cells that did not have lipid-soluble components in their cell walls, was measured. The results (Fig. 8) showed that when the final concentration of GA reaches was 5 μg mL^−1^, a large degree decrease of GFP fluorescence in *E. coli* occurred. When the final concentration of GA was above 10 μg mL^−1^, it could completely quench the GFP fluorescence in *E. coli*. However, if the lipid-soluble components were not removed from the *E. coli* bacteria cell wall, even the final concentration of GA at 500 μg mL^−1^ could not quench the GFP fluorescence (Fig. 1-a). These results showed that the lipid-soluble components in Gram-negative bacteria cell walls could intercept GA.

## Discussion

In this study, *E. coli* DH5α, *E. coli* O157: H7, *P. putida* KT2440, *P. aeruginosa* PAO1, *R. solanacearum, Rhodococcus* RHA1, *S. thermophilus* ND03, *S. aureus* and other common strains were used to study the antibacterial activity of GA (C15:1), and GA was found to have significant antibacterial activity against Gram-positive bacteria but little effect on the growth of Gram-negative bacteria. A relatively strong selective antibacterial mechanism of GA was observed. The MIC value of *B. amyloliquefaciens* SQR9 was the smallest among all of the tested Gram-positive bacteria. However, its MBC value (60 μg mL^−1^) was the largest among all of the tested Gram-positive bacteria. These results might be caused by small amount of endospores that were generated when *B. amyloliquefaciens* SQR9 was cultured. Endospores had relatively strong resistance, and could withstand higher concentrations of GA without being killed. Therefore, *B. amyloliquefaciens* SQR9 had significantly higher MBC values than other Gram-positive bacteria. The antibacterial activity of GA has been reported these years. Himejima and Kubo [12] found that 2-hydroxy-6-(8-pentadecenyl) salicylic (another name of ginkgolic acid C15:1) showed lower MICs (about 10 μg mL^−1^) against Gram-positive bacteria and higher MICs (>100 μg mL^−1^) against Gram-negative bacteria. Choi et al. [23] also showed that GA (C15:1) had significant antibacterial activity against 18 g-positive vancomycin-resistant. The results of the present study are consistent with above studies.

Additional studies on antibacterial mechanisms using GFP fluorescence-labeled Gram-positive bacteria *B. amyloliquefaciens* SQR9 and GFP-labeled Gram-negative bacteria *E. coli* DH5α and *P. putida* KT2440 showed that GA (C15:1) could significantly affect GFP fluorescence in the cells of Gram-positive *B. amyloliquefaciens* SQR9-gfp, whereas it had no significant effect on GFP fluorescence in the cells of Gram-negative bacteria *E. coli* DH5α-gfp and *P. putida* KT2440-gfp. The green fluorescent protein (GFP) has been widely used as a highly useful tool in the fluorescence studies of living cells, which is found in cell cytoplasm of jellyfish and is an extremely stable protein with 238 amino acids [25, 26]. The fluorescence produced by GFP was caused by its protein conformation. In general, as long as the protein conformation of GFP did not change, the fluorescence would not decay or disappear. Previous reports showed that GA and sumac acids, which had a similar structure, could affect the activity of numerous enzymes, including protein phosphatase, lipoxygenase and histone acetyltransferase [27–29]. In addition, GA affected in vivo regulation mechanism of small ubiquitin-related modifier (SUMO) and altered protein conformation, thereby affecting protein expression [30].

According to the above test, we suggested that the mechanism by which GA (C15:1) decayed GFP fluorescence was through conformation changes in the GFP protein. In addition, the mechanism by which GA promoted antibacterial activity against Gram-positive bacteria was through conformational changes of the proteins in the bacteria that inactivated the proteins and inhibited the growth of Gram-positive bacteria.

The results of crude cell lysate experiments showed that GFP fluorescence decay might be related to the interaction between GFP and GA. The GFP fluorescence in both Gram-negative and Gram-positive bacteria crude lysates was quenched by GA in a short period of time, which indicated that GFP fluorescence would be quenched as long as it had contact with GA and was not related to the microorganism tagged with GFP. Because the structure between Gram-negative and Gram-positive bacteria was similar and results showed that peptidoglycan in Gram-positive bacteria could not prevent GA (C15:1) from entering the cell, we suggested that the peptidoglycan structure of Gram-negative bacteria also could not block GA (C15:1) from entering the cell. In the protoplast experiment, a small amount of GA molecules could enter the cells after the peptidoglycan structure in *E. coli* cell wall was destroyed by lysozyme, which might be the result of the action of lysozyme. After the peptidoglycan structure was destroyed by lysozyme, pores might be present on the surface of the peptidoglycan layer that allowed GA molecules to pass through. However, only a small amount of GA molecules could enter the cells because the number of pores generated on the surface of the peptidoglycan layer was low, and the surface of Gram-negative bacteria was still covered by a large amount of lipids (including lipopolysaccharides and phospholipids), which could intercept a large amount of GA molecules. In order to further confirm lipid-soluble components in the cell wall of Gram-negative bacteria intercept the majority of GA molecules, the studies use high resolution electron microscopy to observe membrane change or other methods to study transport of GA through membrane will be carried out.

Some studies demonstrated that GA markedly inhibited the biofilm formation of *S. mutans* and *Escherichia coli* O157:H7, and disrupted biofilm integrity [24, 31]. Therefore, we speculate that the GA may affect the secondary metabolism of Gram-positive and Gram-negative bacteria. Due to the secondary metabolism of bacteria, such as the formation of biofilm, fluorescence formation and synthesis of antibiotics are regulated by quorum-sensing, further studies on this section will be investigated.

## Conclusions

GA (C15:1) has a relatively strong selective antibacterial mechanism, which significant antibacterial activity against Gram-positive bacteria but little effect on the growth of Gram-negative bacteria. Additional studies on antibacterial mechanisms showed that GA (C15:1) could inhibit the activities of the selected proteins to a certain degree, and non-selectively induce protein conformational changes. GA (C15:1) also inhibit DNA replication in vivo as well as RNA transcription and protein synthesis. Thus, we suggested that the mechanism by which GA (C15:1) promoted antibacterial activity against Gram-positive bacteria was through conformational changes of the proteins in the bacteria that inactivated the proteins and inhibited the growth of Gram-positive bacteria. The research results indicated that lipid-soluble components (including lipopolysaccharide and phospholipids) in the cell wall of Gram-negative bacteria intercepted the majority of GA molecules, whereas the peptidoglycan layer in the cell wall showed a reduced capacity to intercept GA molecules.

## Methods

### Media and reagents

Lysogeny broth (LB) medium (g L^−1^) was composed as follows: peptone 10.0 g L^−1^, yeast extract 5.0 g L^−1^, NaCl 10.0 g L^−1^, pH 7.2, (solid, addition of 1.5% agar), and deionized water 1000 mL, which was sterilized at 121 °C for 20 min.

Proteinase K, lysozyme, ampicillin (Amp), kanamycin (Km), gentamicin (Gm), isopropyl-β-D-thiogalactopyranoside (IPTG), o-nitrophenyl β-D-galactopyranoside (ONPG), and o-nitrophenol (ONP) were purchased from Shanghai Sangon Biotech (Sangon Biotech, Shanghai, China). GA C15:1 standard was purchased from Shanghai Tauto Biotechnology (Shanghai, China). Other chemical reagents were analytical grade.

### Strains

*Escherichia coli* DH5α (*E. coli* DH5α) (ATCC53338), *E. coli* O157: H7 (*E. coli* O157: H7) (ATCC43895), *Pseudomonas putida* KT2440 (ATTC47054), *Pseudomonas aeruginosa* PAO1 (ATCC15692), *Ralstoniasolanacearum* (ATCC11696)*, Rhodococcusjostii* RHA1 [32], *Streptococcus thermophilus* ND03 [33], and *S. aureus* (ATCC25923) were from our laboratory.

*Bacillus amyloliquefaciens* SQR9 (CGMCC 5808; China General Microbiology Culture Collection Center) [34], *E. coli*-gfp (*E. coli* DH5α-gfp), *P. putida* KT2440-gfp, and *B. amyloliquefaciens* SQR9-gfp were provided by the Environmental Microbiology Lab at the College of Resources and Environment, Nanjing Agricultural University.

### Determination of antibacterial activity of GA

A conventional broth-dilution method was adopted [35]. GA and salicylic acid were dissolved into dimethylsulfoxide (DMSO) to prepare stock solutions with different concentrations, respectively. The final concentrations of the drug (GA or salicylic acid) in the medium (500 μg mL^−1^, 250 μg mL^−1^, 100 μg mL^−1^, 80 μg mL^−1^, 60 μg mL^−1^, 40 μg mL^−1^, 20 μg mL^−1^, 10 μg mL^−1^, 5 μg mL^−1^, 2 μg mL^−1^, 1 μg mL^−1^, 0.5 μg mL^−1^ and 0.1 μg mL^−1^) were obtained. 10 μL stock solutions were added to 3 mL liquid LB medium that was inoculated with bacteria. All of the tests were incubated at 200 rpm. *E. coli* was cultured at 37 °C for 2 d, whereas other bacteria were cultured at 30 °C for 2 d. The lowest concentration without turbidity was defined as the minimum inhibitory concentration (MIC) of that substance. The medium without turbidity (50 μL) was inoculated with 3 mL of fresh LB liquid medium, and cultured in a shaker. *E. coli* was cultured at 37 °C for 2 d, and all other bacteria were cultured at 30 °C for 2 d. The lowest concentration without turbidity was defined as the minimum bactericidal concentration (MBC) of that substance. Same concentration of DMSO without GA and salicylic acid was added in control groups.

### Measurement of fluorescence decay of GFP in bacteria

Single colonies of *E. coli* DH5α-gfp, *P. Putida* KT2440-gfp and *B. amyloliquefaciens* SQR9-gfp were extracted from solid LB plates, inoculated in liquid LB medium, and then 50 μg mL^−1^ Amp, Gm and Km was added to the medium to maintain the normal replication of plasmids in each strain. The medium was then centrifuged, and the bacteria were collected. The supernatant was discarded, and phosphate buffer was added to the collected bacteria to obtain the bacteria concentration of 10^8^ CFU mL^−1^. GA was then dissolved in DMSO to prepare stocks with different concentrations.

1. 10 μL of GA stock solution at different concentrations was added to 1 mL of bacteria solution. The final concentrations of GA in the solution were 500 μg mL^−1^, 250 μg mL^−1^, 100 μg mL^−1^, 50 μg mL^−1^, 25 μg mL^−1^, 10 μg mL^−1^ and 5 μg mL^−1^, respectively. 10 μL of DMSO without the drug was used as a control. All of the tests were incubated at 30 °C for 1 min before GFP fluorescence was measured. The morphology of the bacteria was monitored under a scanning electron microscopy. For strains of *E. coli* DH5α-gfp and *P. Putida* KT2440-gfp, the incubation was extended to 4 h and additional samples were collected to measure GFP fluorescence. The morphology of the bacteria was measured using a scanning electron microscopy. Bacteria GFP fluorescence was initially observed by the naked eye using an LB16 Maestrogen UltraSlim no-damage blue LED transilluminator. Measurements of bacterial GFP fluorescence were performed using a Spectra ax M5 multifunctional microplate reader. The samples’ fluorescence intensity was measured when the excitation wavelength was 488 nm and the emission wavelength was 509 nm. The blank was the *E. coli* bacteria solution without GFP.
2. 1 mL of bacteria solution was lysed by sonication. The broken bacteria were centrifuged at 12,000 × g, 4 °C for 20 min, and then the supernatant was collected. GA stock solution was added to the supernatant. The final concentrations of GA in the bacteria solution were 500 μg mL^−1^, 250 μg mL^−1^, 100 μg mL^−1^, 50 μg mL^−1^, 25 μg mL^−1^, 10 μg mL^−1^ and 5 μg mL^−1^, respectively. 10 μL of DMSO (without the drug) was used as a control. All of the tests were incubated at 30 °C for 1 min before GFP fluorescence was measured. The control was the crude enzyme solution of the corresponding bacteria without GFP.

### Effect of GA on the activities of a variety of proteins

#### Effect of GA on the activity of Taq DNA polymerase

Stock solutions at different concentrations were prepared by dissolving GA in DMSO. GA stock solutions (0.5 μL) at different concentrations were then added into each PCR reaction system. The final concentrations of GA in the reaction system were 25 μg mL^−1^, 10 μg mL^−1^, 5 μg mL^−1^ and 1 μg mL^−1^, respectively. 0.5 μL of DMSO (without the drug) was as a control. The PCR reaction conditions were adopted the reference of Chester and Marshak [36]. After the reaction was finished, 3 μL of PCR product was analyzed on an appropriate agarose gel. GelRed nucleic acid dye was used to stain the gel.

#### Effect of GA on the activity of restriction enzymes

Three common restriction enzymes (Kpn I, Hind III and EcoR I) were selected as the targets. GA was dissolved in DMSO to prepare stock solutions with different concentrations. A single colony of *E. coli* that carried the pUC19 plasmid, was extracted and inoculated in 3 mL of LB medium containing Amp, which was then cultured at 37 °C overnight with vigorous shaking. 41.5 μL of pUC19 (200 ng μg^−1^), 5 μL of 10 × restriction enzyme buffer, 3 μL of corresponding restriction enzyme, and 0.5 μL of GA stock solution with different concentrations were mixed. The final concentrations of GA in the reaction system were 25 μg mL^−1^, 10 μg mL^−1^, 5 μg mL^−1^ and 1 μg mL^−1^, respectively. 5 μL of DMSO (without the drug) was used as a control. All of the components in the reaction systems were digested at 37 °C for 4 h, and then the temperature was increased to 75 °C for 15 min to stop the enzyme digestion. Electrophoresis was then carried out to examine the enzyme digestion.

#### Effect of GA on the activity of superoxide dismutase

According to the method by Beauchamp et al. [37], GA was dissolved in DMSO to prepare stock solutions with different concentrations. 80 μmol L ^-1^riboflavin, 77 μmol L ^-1^ nitro blue tetrazolium (NBT), 13 mmol L^−1^ methionine, 0.1 mmol L^−1^ EDTA, 20 μL superoxide dismutase (SOD) enzyme solution (1 μg mL^−1^), and 5 μL of the different concentrations of GA stock solution (final concentrations of GA in the reaction system of 25 μg mL^−1^, 10 μg mL^−1^, 5 μg mL^−1^ and 1 μg mL^−1^) were mixed. 5 μL of DMSO (without the drug) was used as a control. After the samples were exposed to light at 4500 Lux light intensity for 15 min, the reaction was stopped by shielding the light. The optical density (OD)_560_ values were measured. One active unit (U) occurred when NBT was inhibited by 50%, and enzyme activity = (△ A × N × 60)/(W × T × V × 50%), where △ A represented the difference in OD values between the control and sample, N represented the total volume of enzyme solution, W represented the protein mass, T represented the light reaction time and V represented the volume of enzyme solution added. Enzyme activity was represented as OD_560_ • mg^−1^ (pro) • min^−1^.

#### Effect of GA on the activity of β-galactosidase

2 μg mL^−1^ the enzyme β-galactosidase was prepared in 10 mM pH 7.0 phosphate buffer. GA was dissolved in DMSO to prepare the stock solutions with different concentrations. Enzyme solution (1 mL) was incubated at 37 °C for 5 min, then 1 mL of phosphate buffer (pH 7.0) containing 20 mM ONPG preheated to 37 °C was added, finally 5 μL of the different concentrations of GA stock solution were added. The final concentrations of GA in the reaction system were 25 μg mL^−1^, 10 μg mL^−1^, 5 μg mL^−1^ and 1 μg mL^−1^, respectively. 5 μL of DMSO (without the drug) was used as a control. All of the samples were incubated in a 37 °C for 10 min, and then 3 mL of 0.5 mol L^−1^ Na_2_CO_3_ was added to stop the reaction. The OD value at 420 nm was read. One enzyme unit was defined as the amount of enzyme required to release 1 μmol of ONP per minute at 37 °C.

### Inhibition of isotopic precursor incorporation

According to the method by Aspedon and Groisman [21], [^3^H] TdR, [^3^H] UR and [^3^H] Tyr were incorporated into precursors to investigate effect of GA (C15:1) on the biosynthesis of DNA, RNA and *B. amyloliquefaciens* SQR9 protein. The logarithmic growth phase SQR9 culture was diluted with sterile water. In a 96-well plate, 0.9 mL of bacterial suspension (OD_600_ = 0.1) was added to each well. The isotope-labeled precursor (final concentration was 0.5 μCi mL^−1^) and 5 μL of the different concentration of GA (C15: 1) were also added into the well. The final concentrations of GA in the reaction system were 25 μg mL^−1^, 10 μg mL^−1^ and 5 μg mL^−1^, respectively. 5 μL of DMSO (without the drug) was used as a control. All of the treatments were conducted at 37 °C in a shaker. When examining effect of GA (C15:1) on the synthesis of DNA and RNA, culture time for bacteria was limited to one generation. After growing the culture for 30 min, the culture was centrifuged at 12,000 × *g*, 4 °C for 5 min to harvest the bacteria pellet. Because protein synthesis was relatively slow, the bacteria culture time was extended to 2 h. OD_600_ was also adjusted so that the cultures had the same bacteria concentration. The bacteria pellet was washed three times with phosphate buffer and then placed in an oven overnight to dry it. Scintillation solution was directly added to the Eppendorf tubes containing bacteria, and the tubes were then transferred to a scintillation counter (LS3801, Beckman) to determine the counts per minute (CPM) values. The average CPM values of the experimental group and control group were compared. The incorporation inhibition rate was calculated. Incorporation inhibition rate = control group CPM - experimental group CPM/control group CPM × 100%.

### Effect of GA (C15:1) on GFP fluorescence in the protoplasts of *E. coli*

*E. coli* protoplasts were prepared according to the method described by Weiss [38]. GA was dissolved in DMSO, and its stock solutions with different concentrations were prepared. *E. coli* protoplasts (1 mL) were diluted with sucrose-magnesium-maleate (SMM) buffer (protoplast number > 10^7^ CFU mL^−1^), and 5 μL of the different concentration GA stock solutions were added. The final concentrations of GA in the reaction system were 500 μg mL^−1^, 250 μg mL^−1^, 100 μg mL^−1^, 50 μg mL^−1^, 25 μg mL^−1^, 10 μg mL^−1^ and 5 μg mL^−1^, respectively. 5 μL of DMSO (without the drug) was used as a control. All the tests were incubated at 30 °C for 1 min, and then the samples were analyzed for GFP fluorescence. GFP fluorescence was initially observed by the naked eye using an LB-16 Maestrogen UltraSlim no-damage blue LED transilluminator. GFP fluorescence was measured with a Spectra ax M5 multifunctional microplate reader. The samples’ fluorescence intensity was measured when the excitation wavelength was 488 nm and the emission wavelength was 509 nm. The blank was *E. coli* protoplast solution without GFP.

### The interception of GA by the lipid-soluble component in *E. coli* cell walls

*E. coli* DH5α-gfp was cultured in 100 mL of liquid LB medium at 37 °C, 200 rpm overnight. After centrifugation at 12,000 × *g* for 5 min, the supernatant was discarded to collect the bacteria. The bacteria pellet was then re-suspended in the same volume of phosphate buffer. The half was retained for the experiments and the left half was centrifuged at 12,000 × *g* for 5 min. The supernatant was then discarded. A small volume of phosphate buffer was used to re-suspend the bacteria pellet, and then 10 mL of 70% ethanol solution was added, mixed well, and kept at room temperature for 30 s before centrifuging at 12,000 × *g* for 1 min. The supernatant ethanol solution was then discarded. Phosphate buffer (50 mL) was used to re-suspend the bacteria pellet, which was centrifuged at 12,000 × *g* for 5 min. The supernatant was then discarded. Finally, 50 mL of phosphate buffer was used to re-suspend the bacteria pellet for the experiments.

GA was dissolved in DMSO, and its stock solutions with different concentrations were prepared. GA stock solutions (5 μL) at different concentrations were added to 1 mL of bacteria solution before/after ethanol solubilization of the lipids. The final concentrations of GA in the reaction system were 500 μg mL^−1^, 250 μg mL^−1^, 100 μg mL^−1^, 50 μg mL^−1^, 25 μg mL^−1^, 10 μg mL^−1^ and 5 μg mL^−1^, respectively. 5 μL of DMSO (without the drug) was used as a control. All the tests were incubated at 30 °C for 1 min, and then the samples were examined for GFP fluorescence. GFP fluorescence was initially observed by the naked eye using an LB-16 Maestrogen UltraSlim no-damage blue LED transilluminator. GFP fluorescence was measured using a Spectra ax M5 multifunctional microplate reader. The samples’ fluorescence intensity was measured when the excitation wavelength was 488 nm and the emission wavelength was 509 nm. The blank was *E. coli* bacteria solution without GFP (after ethanol solubilization).

### Statistical analyses

For all the experiments throughout the study, we collected three individual tubes from LB plates, and each tube was performed in triplicate. In analysis, comparisons were carried out using the fluorescent means of each triplicate, then, finally each bar in the gram represents means of three individual tubes. The data of each strain was analyzed using one-way ANOVA (i.e., *B. amyloliquefaciens* SQR9-gfp *E. coli* DH5α-gfp and *P. putida* KT2440-gfp), with final concentration of GA as the fixed factors (*P* < 0.05). The experimental data were log transformed to meet the homogeneity of variance or a normal distribution of residuals. All statistical analyses were conducted using SPSS 13.0 (SPSS, Chicago, IL, USA).

## Abbreviations

[^3^H] TdR: (Methy-^3^H) thymine
[^3^H] Tyr: ^3^H-tyrosine
[^3^H] UR: ^3^H-uridine
Amp: Ampicillin
CFU: Colony-Forming Units
CK: Control check
CPM: Counts per minute
DMSO: Dimethylsulfoxide
DNA: Deoxyribonucleic acid
EDTA: Ethylenediaminetetraacetic acid
GA: Ginkgo acid
GFP: Green fluorescent protein
Gm: Gentamicin
IPTG: Isopropyl-β-D-thiogalactopyranoside
Km: Kanamycin
LB: Lysogeny broth
LED: Light emitting diode
MBC: Minimum bactericidal concentration
MIC: Minimum inhibitory concentration
NBT: Nitro blue tetrazolium
OD: Optical density
ONP: O-nitrophenol
ONPG: O-nitrophenyl-β-D-galactopyranoside
PCR: Polymerase chain reaction
RNA: Ribonucleic acid
SMM: Sucrose-magnesium-maleate
SOD: Superoxide dismutase
